# A Swedish Nationwide prospective study of oncological and reproductive outcome following fertility-sparing surgery for treatment of early stage epithelial ovarian cancer in young women

**DOI:** 10.1186/s12885-020-07511-y

**Published:** 2020-10-19

**Authors:** Gry Johansen, Pernilla Dahm-Kähler, Christian Staf, Angelique Flöter Rådestad, Kenny A. Rodriguez-Wallberg

**Affiliations:** 1 Department of Oncology-Pathology, Karolinska Institutet, Stockholm, Sweden; 2 Department of Gynecology, Division of Gynecology and Reproduction, Karolinska University Hospital, Stockholm, Sweden; 3 Department of Obstetrics and Gynecology, Sahlgrenska University Hospital, Gothenburg, Sweden; 4 Institute of Clinical Sciences, Sahlgrenska Academy at University of Gothenburg, Gothenburg, Sweden; 5 Regional Cancer Center Western Sweden, Sahlgrenska University Hospital, Gothenburg, Sweden; 6 Department of Women’s and Children’s Health, Division of Obstetrics and Gynecology, Karolinska Institutet, Stockholm, Sweden; 7 Department of Reproductive Medicine, Division of Gynecology and Reproduction, Karolinska University Hospital, Novumhuset Plan 4, 141 86 Stockholm, Sweden

**Keywords:** Fertility preservation, Fertility-sparing surgery, Early-stage epithelial ovarian cancer, Overall survival, Assisted reproductive technology treatment, Obstetrical outcome

## Abstract

**Background:**

Epithelial ovarian cancer (EOC) is rare in women of reproductive age and fertility-sparing surgery (FSS) may be applied in early stages. The purpose of this study was to investigate the safety and efficacy of FSS for treatment of EOC.

**Methods:**

The Swedish nationwide population-based Quality Register for Gynecological Cancer was used to identify all women 18–40 years of age diagnosed with stage I EOC between 2008 and 2015. Detailed data on surgery, staging, histopathology, and follow-up were extracted and reviewed. Cross-linking of individuals to population-based registries allowed retrieval of data on obstetrical and reproductive outcomes after FSS. Disease-free survival (DFS) and overall survival (OS) rates were compared (Kaplan-Meier method) between women who underwent FSS vs. radical surgery (RS).

**Results:**

In total 83 women were identified; 36 who had FSS performed and 47 RS. The 5-year OS rate was 92% and no statistical differences between DFS or OS were found between women treated by FSS or RS. The recurrence rate after RS was 13% compared to 6% after FSS. Recurrences were more frequently found in women with stage IC tumor or with histologic subtypes with more aggressive behavior. In the FSS cohort, nine women gave birth to 12 healthy children, all delivered at fullterm. Only one women had received assisted reproductive technology treatment.

**Conclusion:**

In this nationwide population-based cohort study natural fertility was maintained after FSS. Specific histologic subtypes showed greater prognostic impact on the oncological outcome than the use of FSS. Recurrences occurred after FSS, but none in the uterus, which questions the need of hysterectomy in young women with EOC.

**Trial registration:**

This article reports the results of a healthcare intervention using the data prospectively registered in the Swedish population-based registries including the Quality Register for Gynecological Cancer, the National Death Register, the Swedish Medical Birth Register, and the National Quality Register for Assisted Reproduction.

## Background

Epithelial ovarian cancer (EOC) is rare, but may occur, in women of reproductive age. Fertility preservation is an important issue of high relevance for quality of life in young women presenting with cancer [1]. As EOC’s histological subtypes differ in their malignancy potential, there is no current international consensus about the substages or histologic subtypes and grades at which fertility-sparing surgery (FSS) is considered as safe as radical surgery (RS) for the specific treatment of young women of reproductive age. Some authors recommend restricting the practice of FSS to patients with stage IA and with grade 1 tumors [2] or with unilateral grade 1 or 2 tumors [3–8], whereas others propose FSS to all patients with stage IA–IC tumors [9–13]. The real impact of FSS on patient prognosis remains under debate, since most relapses are distant. It has been suggested that the aggressiveness of the disease, rather than the sparing of the ovary and uterus, is what ultimately affects the risk of distant metastases [7, 10, 12, 14].

Most studies reporting FSS for EOC have involved small cohorts [7], and although a few multicenter studies are available, there is lack of prospective studies [5, 14, 15]. This is mainly due to the very small number of women diagnosed with EOC worldwide during fertile age. Difficulties in finding a comparable group undergoing RS for control, and lack of detailed information on surgical staging, tumor grade evaluation, and histological subtypes are important shortcomings of the available studies. Further on, although the purpose of FSS is to preserve fertility, data on obstetrical outcomes and also on the use of assisted reproductive technology (ART) for treatment of infertility in women treated with FSS for ovarian cancer are scarce.

We hereby report a prospective study of FSS vs RS for treatment of young women with early stage EOC, using the Swedish population-based healthcare registries. Our research methods have been previously validated for the investigation of safety and efficacy of FSS in young women presenting with non-epithelial types of ovarian cancer [16]. In the current study, the linking of identified individuals among registries such as the Quality Register for Gynecological Cancer, the National Death Register, the Swedish Medical Birth Register, and the National Quality Register for Assisted Reproduction, allowed us to conduct a nationwide investigation extracting clinical data, oncological follow-up and obstetrical outcomes of young women treated with FSS. Our aim with this study is to report on the safety and efficacy of FSS for treatment of early stage EOC, and compare oncologic outcomes with women undergoing RS.

## Methods

The Swedish Quality Register for Gynecological Cancer (SQRGC) was used to identify the study cohort, which included all women aged 18–40 years, undergoing FSS or RS for stage I EOC in Sweden between 2008 and 2015. The methods used have been validated in a previous investigation of oncologic and reproductive outcomes of women with ovarian cancers of non-epithelial type and thoughtfully described [16]. Briefly, The SQRGC, launched in 2008, captures prospectively and consecutively collected clinical information, surgical details, and oncological variables and outcomes that are continuously updated ensuring follow-up, together with mortality data. The validity of the recorded data and the agreement between variables among the SQRGC and the National Swedish Cancer Registry (NCR), which collects compulsorily cancer incidence in Sweden is close to 100% [17]. To ensure lifelong follow-up and date of death, a linkage to the National Death Registry was done, using the 10-digit personal identification number assigned to all Swedish citizens. To obtain obstetrical data in cases where the women have given birth after FSS a linkage to the Swedish Medical Birth Registry, which includes data on all deliveries in Sweden and information from standardized prenatal, delivery, and neonatal care, was done. Information on provision of assisted reproductive technology (ART) treatment, such in vitro fertilization (IVF), was obtained by further linkage of the FSS cohort to the National Quality Register for Assisted Reproduction (Q-IVF). The Swedish Q-IVF was founded in 2007 and it includes data on all ART treatments given in Sweden with a coverage of 100% of treatments (https://www.socialstyrelsen.se/register/registerservice/nationellakvalitetsregister/q-ivfnationelltkvalitetsregist). In Sweden women up to an age of 40 years can receive fertility treatments free of charge within the health care system. During the study period the women could not apply for fertility treatment if they lack a partner, which changed only with a change in the law in 2017.

Tumor classification was registered in the SQRGC, according to World Health Organization criteria [18], and the classification updated from 2014. The Silverberg classification system [19] was used to grade tumors, which were defined according to the 2006 International Federation of Gynecology and Obstetrics (FIGO) metrics. High-grade serous tumors, clear cell tumors and endometrioid tumors grade 3, were classified as tumors with highly aggressive potential. The FIGO 2014 classification system was used for clinical staging [20], and stage IC tumors were further classified as IC1, IC2 or IC3.

Similarly to as in our previous investigation of ovarian cancers of non-epithelial type, FSS was defined as the preservation of the uterus and at least part of one ovary [16]. RS was defined as hysterectomy with bilateral oophorectomy. Patients were censored at the time of data retrieval (July 27, 2017), if they were known to be alive. Survival estimates included overall survival (OS), calculated from the date of diagnosis to either the date of death from any cause or the date of data retrieval and disease-free survival (DFS), defined as the time from diagnosis to either the first appearance of relapse or the date of death from any cause. Patients known to be free of relapse were censored at the time of data retrieval. All medical records were reviewed for detailed data on the relapses and in cases were the registration records were incomplete. The Regional Ethics Committee of Stockholm approved the study (Dnr 2016/1161–31/2).

### Statistical analysis

Comparison of continuous variables was performed using the Student’s t-test and ANOVA. Fisher’s exact test was used for categorical variables, as appropriate for each category size. A 5% level of significance, 2-sided, was used for all comparisons. Stata statistical software (Macintosh version 13.1) was used for the statistical analysis [21]. Oncologic outcomes DFS and OS were analyzed using the Kaplan-Meier method.

## Results

### Flowchart and demographics

**Fig. 1:**
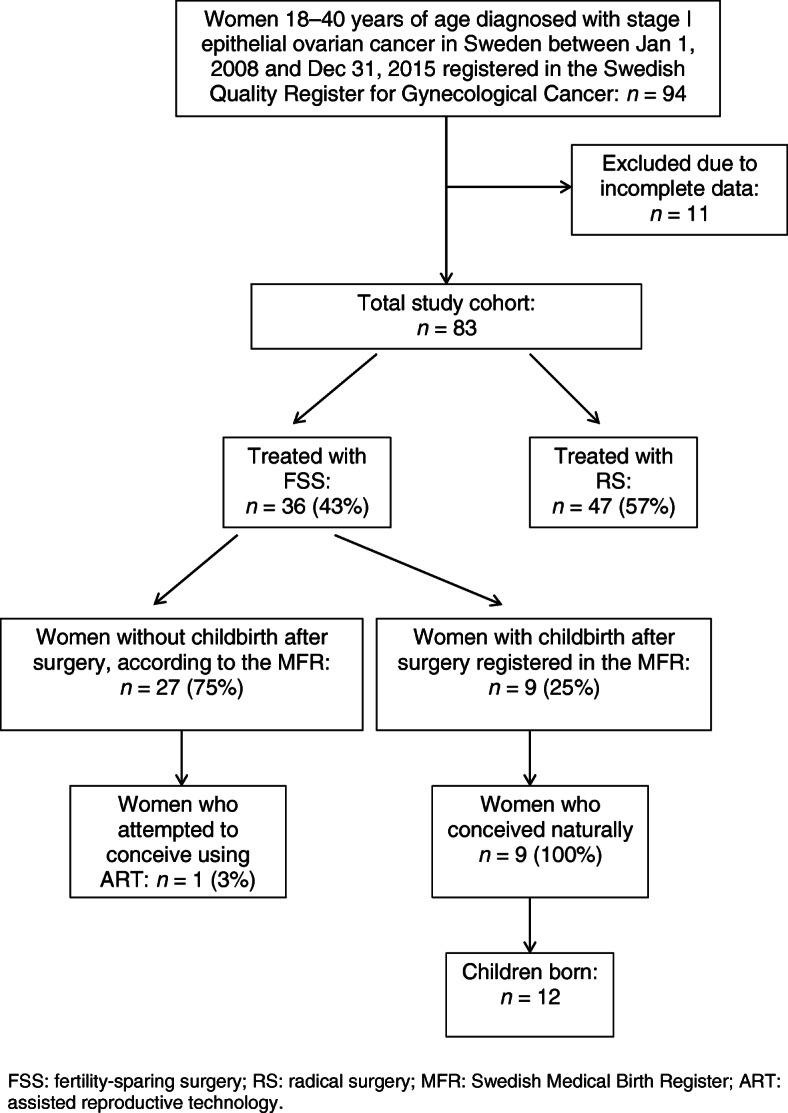
Flow chart of the study population. FSS: fertility-sparing surgery; RS: radical surgery; MFR: Swedish Medical Birth Register; ART: assisted reproductive technology

During the study period 83 women aged 18–40 years diagnosed with EOC and with complete data, were identified in the SQRGC. A patient flow chart is shown in Fig. 1. There were 11 patients who were excluded due to incomplete data. They did not differ from the study cohort regarding age, tumor type, or stage. Histologic reviews were performed in 90% (75/83) of the cases by a pathologist with expertise in gynecologic oncology and the cases discussed at a multidisciplinary conference. FSS was performed in 43% (*n* = 36) of the 83 patients, in all cases a unilateral oophorectomy was performed. These women were significantly younger than the 47 patients who underwent RS (*p* < 0.001), and had a lower previous parity (*p* < 0.001). The women undergoing RS were more often diagnosed in stage IC—62% (29/47) vs. 25% (9/36) (*p* = 0.002). They also more often had tumors with highly aggressive potential, were more often surgically staged with lymph node dissections, and were more likely to receive adjuvant chemotherapy (*p* = 0.003) (Table 1). All patients were diagnosed in either stage IA or stage IC, there were no cases of stage IB during the study period. There were no differences in follow-up between women who underwent FSS and RS, 63 months (16–111) and 64 months (21–112), respectively.

**Table 1:** Demographics, clinical and surgical data, oncological treatment, and outcomes for women 18–40 years old diagnosed with epithelial ovarian cancer in Sweden 2008–2015

	Treated with FSS	Treated with RS	***P-***value
**N** (%)	36 (43)	47 (57)	
**Mean age**, years (range)	29 (19–39)	37 (26–40)	< 0.001
**Previous parity**, n (%)			< 0.001
0	27 (75)	16 (34)	
1	6 (17)	7 (15)	
2	2 (6)	17 (36)	
≥3	0	4 (9)	
**FIGO stage**, n (%)			0.002
IA	27 (75)	18 (38)	
IB	–	–	
IC	9 (25)	29 (62)	
*IC1*	*6*	*19*	
*IC2*	*3*	*6*	
*IC3*	*0*	*2*	
*IC unspecified*	*0*	*2*	
**Histologic type**, n (%)			
Serous, high-grade	2 (6)	6 (13)	
Serous, low-grade	1 (3)	2 (4)	
Mucinous	18 (50)	12 (26)	
Endometrioid	10 (28)	12 (26)	
*Grade 1*	*6*	*4*	
*Grade 2*	*2*	*3*	
*Grade 3*	*1*	*3*	
Clear cell	3 (8)	13 (28)	
*Other* ^*a*^	*2 (6)*	2 (4)	
**Surgical mode**,^**b**^ n (%)	27 (75)	41 (87)	0.241
Open	6 (17)	3 (6)	
Laparoscopy Robot	3 (8)	3 (6)	
**Type of surgery**, n (%)			
Primary	17 (47)	20 (43)	0.824
Re-staging	19 (53)	27 (57)	
**Type of staging procedure**, n (%)			
Cytology	33 (92)	39 (83)	
Peritoneal biopsies	30 (83)	39 (83)	
Oment biopsy	31 (86)	45 (96)	
LN pelvic	8 (22)	24 (51)	
LN paraaortic	8 (22)	22 (47)	
**Adjuvant chemotherapy**, n (%)	14 (39)	34 (72)	0.003
**Histologic review,**^**c**^ n (%)	33 (92)	42 (89)	1.0
**Recurrence**, n (%)	2 (6)	6 (13)	
**Died of disease**, n (%)	1 (3)	2 (4)	
**Follow-up**, months, mean (range)	63 (16–111)	64 (21–112)	0.708

^a^ Mixed type, adenocarcinoma unspecified; ^b^ If primary surgery is laparoscopic and re-staging laparotomy, patient is classified as laparotomy; ^c^ Histologic review by a pathologist with expertise in gynecologic oncology; *FSS* Fertility-sparing surgery, *RS* Radical surgery, *LN* Lymph node

### Oncological outcomes

**Fig. 2:**
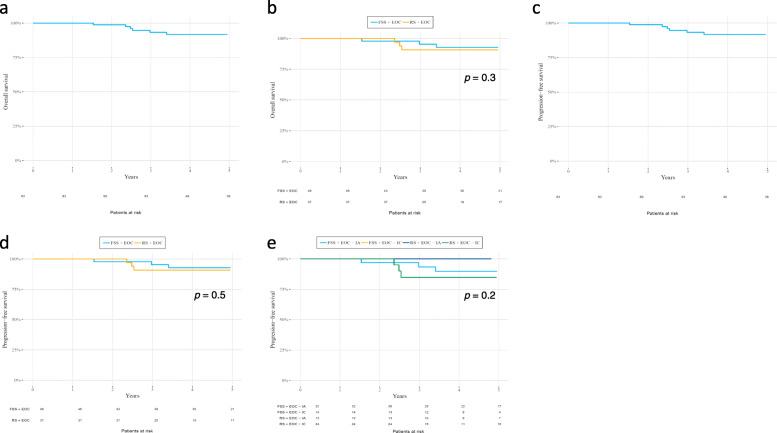
**a** Five-year overall survival (OS) rate for women 18–40 years old with stage-I epithelial ovarian cancer (EOC). The 5-year OS rate is 92%. Patients at risk are shown under the curve. **b** Five-year overall survival (OS) rates for women 18–40 years old treated for epithelial ovarian cancer (EOC) with fertility-sparing surgery (FSS) vs. radical surgery (RS). The 5-year OS rate is 97% for FSS and 89% for RS. Patients at risk are shown under the curve. **c** Five-year progression-free survival (PFS) rate for women 18–40 years old with epithelial ovarian cancer (EOC). The 5-year PFS rate is 88%. Patients at risk are shown under the curve. **d** Five-year progression-free survival (PFS) rates for women 18–40 years old treated for epithelial ovarian cancer (EOC) with FSS vs. RS. The 5-year PFS rate for FSS is 93 and 82% for RS. Patients at risk are shown under the curve. **e** Five-year progression-free survival (PFS) rates for women 18–40 years old treated with FSS vs. RS for FIGO stages IA and IC epithelial ovarian cancer (EOC). The 5-year PFS rates are as follows: FSS IA–93%; FSS IC–93%; RS IA–82%; and RS IC–82%. Patients at risk are shown under the curve

A 5-year OS rate of 92% was found in the total cohort (Fig. 2a). The 5-year OS rates comparison between FSS and RS is shown in Fig. 2b, and these were 97 and 89% respectively, there were no significant difference (*p*-value 0.3). In the total cohort the 5-year DFS rate was 88% (Fig. 2c), with 93 and 82% for FSS and RS, respectively (Fig. 2d), (p-value 0.5). According to tumor stage, the 5-year DFS were 93, 93, 82, and 82% for FSS stage IA, FSS stage IC, RS stage IA, and RS stage IC, respectively, there were no significant difference between the groups (p-value 0.3) (Fig. 2e).

### Recurrence and deaths

In total eight recurrences (10%) were diagnosed: three in stage IA, three in IC1, and two in IC2. The recurrence rate per stage was 7% (3/42), 12% (3/25), and 22% (2/9), respectively (Table 2). Recurrence rates were higher for tumors with highly aggressive potential (14%), compared to tumors with lower aggressive potential (7%), regardless of the surgical approach. The highest recurrence rate was seen in tumors with highly aggressive potential treated with RS: five out of 22 recurred (23%). All five had also received adjuvant chemotherapy. On the contrary, there were no recurrences among the six women (three with stage IA and three with stage IC) with highly aggressive tumors treated with FSS. Women with tumors with lower aggressive potential showed in general a lower recurrence rate of 5% (1/22) after RS and 7% (2/30) after FSS. One of the two women who experienced a recurrence after FSS had an ovarian recurrence that was treated successfully with radical surgery including oophorectomy of the remaining ovary, hysterectomy, supracolic oment resection, peritoneal biopsies and cytology from the abdominal cavity. There was no evidence of disease 14 months after that surgery (Table 2).

**Table 2:** Details on recurrences and cause of death in women 18–40 years of age diagnosed with stage I epithelial ovarian cancer between 2008 and 2015

#	FIGO Stage	Histology	Grade	FSS	Adjuvant Chemo	Recurrence	Time to Recur. (months)	Site of Recurrence	Treatment at Recur.	Follow-Up after Recur. (months)	Status	Time from Treatment to Death (months)	Cause of Death
1	IA	Mucinous	–	Yes	No	Yes	18	Bone & CNS	Radiation + chemo	6	DOD	36	DOD
2	IA	LGS	–	Yes	No	Yes	43	Ovary	Surgery	14	NED	–	
3	IC1	Mixed	1	No	No	Yes	24	Pelvic cavity	Surgery + chemo	2.5	NED	–	
4	IC2	HGS	–	No	Yes	Yes	27	Paraaortic LN & pelvic cavity	Surgery + chemo	3	NED	–	
5	IC1	CCC	–	No	Yes	Yes	16	Pelvic cavity	Surgery + chemo	6	AWD	–	
6	IC1	CCC	–	No	Yes	Yes	10	Pelvic LN & pelvic cavity	Chemo	11	DOD	28	DOD
7	IA	HGS	–	No	Yes	Yes	26	Vaginal cuff	Surgery + chemo	10	NED	–	
8	IC2	CCC	–	No	Yes	Yes	4.5	Peritoneal & liver	Chemo	19	DOD	30	DOD
9	IC1	Mucinous	–	No	Yes	No	–	–	–	–	DEAD	72	Lung cancer
10	IA	Mucinous	–	No	No	No	–	–	–	–	DEAD	18	Jejunal cancer
11	IC	Mucinous	–	No	No	No	–	–	–	–	DEAD	30	Unknown
**Total**, mean (range)	IA (36%)IC (64%)						21 (4.5–43)			9 (2.5–19)		36 (28–72)	

*FSS* Fertility-sparing surgery, *HGS* High-grade serous carcinoma, *LGS* Low-grade serous carcinoma, *CCC* Clear-cell cancer, *CNS* Central nervous system, *LN* Lymph node, *NED* No evidence of disease, *AWD* Alive with disease, *DOD* Died of disease

In this cohort of young women presenting with EOC, six women died during the study period: three from EOC and three from other causes (Table 2). Of the three women who died from EOC, two had undergone RS for stage IC1, respectively IC2, clear cell cancers. One of them had undergone a complete staging procedure with systematic lymphadenectomy and both received adjuvant chemotherapy, either with carboplatin alone or combined with paclitaxel, as a part of their primary treatment. The third woman who died from EOC had undergone FSS for stage IA mucinous cancer. She had not received adjuvant chemotherapy as part of her primary treatment (Table 2).

### Reproductive outcomes

In the cohort of women undergoing FSS, nine (25%) had given birth after surgery, with a mean interval of 34 months (14–65) from cancer treatment to pregnancy. The mean age was similar between those who had given birth and those who had not (Table 3). All women who had given birth had a partner. The follow-up time was significant longer in women who had given birth, 75 months (44–111) compared to 56 months (16–99) in women who had not given birth after surgery. Only one woman (3%) in the total cohort of women, who underwent FSS, was registered to have received ART treatment. No women treated for high-grade serous cancer or clear-cell cancer had given birth after surgery.

**Table 3:** Demographics, clinical and tumor characteristics at diagnosis, surgical data, and oncological outcomes for women 18–40 years old who either had or had not given birth after undergoing FSS for epithelial ovarian cancer in Sweden 2008–2015

	Given Birth After FSS	Not Given Birth After FSS	***P-***value
**N** (%)	9 (25)	27 (75)	
**Mean age**, years (range)	28 (23–37)	29 (19–39)	0.626
**Previous parity**, n (%)			
0	5 (56)	22 (81)	0.133
1	3 (33)	3 (11)	
2	1 (11)	1 (4)	
≥ 3	0	0	
**FIGO stage**, n (%)			
IA	5 (56)	22 (81)	0.184
IB	–	–	
IC	4 (44)	5 (19)	
*IC1*	*4*	*2*	
*IC2*		*3*	
*IC3*	*–*	*–*	
**Histologic type**, n (%)			
Serous, high-grade		2 (7)	
Serous, low-grade	1 (11)		
Mucinous	4 (44)	14 (52)	
Endometrioid	4 (44)	6 (22)	
*Grade 1*	*3*	*3*	
*Grade 2*		*2*	
*Grade 3*	*1*	*–*	
Clear cell	0	3	
*Other* ^*a*^	0	2	
**Surgical mode,**^**b**^ n (%)			
Open	6 (67)	21 (78)	0.335
Laparoscopy	3 (33)	3 (11)	
Robot	0	3 (11)	
**Type of surgery**, n (%)			
Primary	4 (44)	13 (48)	
Re-staging	5 (56)	14 (52)	
**Type of staging procedure**, n (%)			
Cytology	8 (89)	22 (82)	
Peritoneal biopsies	9 (100)	27 (100)	
Oment	6 (67)	20 (74)	
LN pelvic	1 (11)	2 (7)	
LN paraaortic	1 (11)	3 (11)	
**Adjuvant chemotherapy**, n (%)	4 (44)	9 (33)	0.693
**Histologic review,**^**c**^ n (%)	8 (89)	25 (93)	1.00
**Recurrence**, n (%)	0	2 (7)	
**Died of disease**, n (%)	0	1 (4)	
**Follow-up**, months, mean (range)	75 (44–111)	56 (16–99)	0.04

A total of 12 children were born to the nine women who had given birth. The mother’s mean age at time of delivery was 30 years (26–38). All deliveries were singleton, occurred at full-term, at a mean gestational age at birth 39 + 6 weeks (37–42). All but one child that was delivered by a planned cesarean section were delivered vaginally. Three of the vaginal deliveries were induced. No congenital malformations were registered and the mean birth weight was 3632 g (2935–4390) (Table 4).

**Table 4:** Obstetrical outcomes for women who underwent FSS for treatment of stage I epithelial ovarian cancer in Sweden 2008–2015

	Patients (N)	Children Born
**N**	9	12
**Age at time of delivery**, mean (range)		30 (26–38)
**Delivery mode**, n (%)		
Vaginal		11 (92)
Planned cesarean section		1 (8)
Unplanned cesarean section		0
**Induced delivery**^*a*^		3 (25)
**Births**, n (%)		
Single		12 (100)
Twins		0
**Gestational age at birth**, mean (range)		39 + 6 (37 + 2–42 + 1)
**Child weight at birth**, g, mean (range)		3632 (2935–4390)
**Child length at birth**, cm, mean (range)		50.8 (48–53)
**Apgar score**, mean (range)		
1 min		8 (7–10)
5 min		9 (9–10)
10 min		10 (10–10)

^a^Ended in vaginal delivery: *n* = 3; *FSS* Fertility-sparing surgery.

## Discussion

Being EOC a relatively uncommon disease in young women, this study adds on to the available data by reporting on both the safety and efficacy of FSS through a population-based prospective study. The 92% OS rate in the total cohort of 83 women should be considered excellent and within the expected range. The 5-year DFS rate of 88% and the total recurrence rate of 10% (8/83) are in accordance with previously published data [3, 6, 10, 15, 22–24].

Following FSS, the recurrence rate was lower (6%) than after RS (13%), which indicates that the prognosis seems not to be compromised by the FSS procedures. This is also in accordance with previously published data [14]. However, the larger recurrence rate after RS could be explained by the larger number of tumors with highly aggressive potential and stage IC tumors in the RS group. Staging procedures with lymph node dissections and adjuvant chemotherapy were found more frequently in the RS group, which is appropriate, considering this cohort’s selection by histology and stage. In agreement with the likelihood of higher recurrence for more advanced stages, the recurrence rate for stage IC2 was 22%, although figures were small. Kajiyama et al. [25] reported a higher risk of peritoneal recurrence in patients in stage IC2 or IC3, compared to stages IA and IC1, regardless of FSS. Other studies have found that patients with stage IC2 or IC3 had a significantly higher risk of recurrence than those with IC1 [7, 22], and those with stage IB or IC were twice as likely to experience a recurrence than those with IA [3]. In addition, grade 3 tumors have been found to be associated with poor prognosis [3, 4, 10, 15, 22] and distant recurrences [4, 15, 22, 23]. RS is unlikely to reduce the risk of recurrence of these tumors, as suggested by some studies [7, 12, 14, 23]. In this study, there was only one recurrence located in the ovary after FSS, which was successfully treated with surgery. Other authors have reported a higher rate of recurrence isolated in the spared ovary, but the majority had been successfully treated [5, 10]. Recurrence in the preserved uterus is rarely described [22]. Hence, it could be an option to spare the uterus in women with a desire for preserved fertility, creating the possibility of future pregnancy using donor eggs or the woman’s own frozen oocytes.

In our cohort, none of the six women presenting with tumors with highly aggressive potential who underwent FSS had had a recurrence, while five of the 22 women (23%) with tumors with highly aggressive potential who underwent RS had had a recurrence. Our data supports the previous finding that a poor prognosis may be related to the natural history of the disease and to the type of cancer, rather than to a specific surgery type [22, 23, 26]. However, since most patients selected for FSS tend to have a better initial prognosis than those treated with RS, it is difficult to perform valid comparisons. It is necessary to consider differences in histology, stage, and age as well as the number of patients in the study and the lack of appropriate comparators [2, 4, 5, 10, 11, 15, 25]. Nonetheless, most authors currently agree that tumor stage is a critical factor in selecting patients for FSS. Complete surgical staging, including systematic lymphadenectomy, is also of importance in early stage EOC [3, 6, 22]. Interestingly, four of the six women in our cohort who died were diagnosed with mucinous cancer, even though only one of those women died of EOC. Although not proven, it could be possible that those cases had an ovarian metastasis from an undiagnosed primary gastrointestinal or lung cancer or were at a more advanced stage at the time of surgery (Table 2).

The highly reliable fertility and obstetric data of our study indicate that the ability to conceive is preserved by using FSS, supporting this conservative treatment as an option for young women with stage I EOC. Furthermore, all women conceived naturally and their obstetrical outcomes were not affected. The conception rate was 25% (9/36), within reported ranges of 13–65% [3, 5, 9, 27, 28]. Unfortunately the pregnancy rate could not be calculated in this study, as we lacked of information on women with active pregnancy wish that attempted to conceive during the study period. However, data from Q-IVF might be used as an indicator of a desire for fertility when infertility was present in the women of this cohort. The use of ART treatment in our FSS cohort was only 3% (1/36). Satoh et al. [5] reported the use of ART treatment to be 9% in a similar population. Similarly to as in our previous study of FSS for treatment of young women with non-epithelial ovarian cancer [16], follow-up time was significantly longer in the cohort of women who gave birth after FSS, compared to those who did not (75 vs. 56 months, respectively; *p* = 0.04). A possible explanation may lie in the fact that women might postpone childbearing due to fear of recurrence, or maybe due to non active desire for pregnancy, even though nearly 5 years had passed since surgery. The mean time from cancer treatment to pregnancy found in this study was 34 months. Time to pregnancy has not been previously reported in any of the available studies on EOC.

A limitation to our study is obviously the low volume of patients of young age diagnosed with early stage EOC. However, our study’s major strengths are the use of a cohort with complete lifelong and nationwide coverage and the access to prospectively and consecutively gathered data of high quality, which has been validated for oncology, fertility and obstetrical outcomes [16]. Aiming at full completeness, data was supplemented with information from the medical records, and the National Death Register to ensure lifelong follow-up. Detailed information on important prognostic variables such as tumor stage (substage IC), grade and surgical procedure were nearly 100% complete in our study, with expert pathology review and multidisciplinary discussion performed in a high percentage of cases. We also had a comparator group of women that underwent RS. The research methods used in this study have been previously validated for the investigation of outcomes following FSS in women treated for non-epithelial types of ovarian cancer in Sweden [16].

The mean follow-up time of 65 months (19–114) may be considered long enough to reduce the risk of underestimating the number of recurrences or overall survival rates. The median time to recurrence or death in the cohort was 21 (4.5–43) and 36 months (28–72), respectively. Although the sample size did not allow subgroup analysis according to histopathological subtypes, the detailed data from our study could be useful for future meta-analysis and may add further valuable information.

## Conclusions

In this prospective investigation using nationwide population-based healthcare registry data, the treatment of stage I EOC through FSS in women of fertile age was not associated with worse survival outcomes than that of women who underwent RS. Women who were treated with RS had worse prognostic factors at the time of diagnosis, such as stage IC tumors and histologic subtypes of highly aggressive potential. Relapses were generally rare in the cohort, but the recurrence rate was higher in the RS group and in women with stage IC tumor or with histologic subtypes with more aggressive behavior, which indicates that disease characteristics may be more important for prognosis than the surgical approach. Fertility was maintained after FSS and the obstetrical outcomes were not affected. FSS may therefore be an option for women of fertile age with early stage EOC, after consultation with a multidisciplinary gynecologic board and discussions with patients and their families.

## Data Availability

The datasets generated and/or analyzed during the current study are not publicly available, but are kept at the Regional Cancer Center Western Sweden. These data might be available from the corresponding author on reasonable request.

## Abbreviations

ART: Assisted reproductive technology
DFS: Disease-free survival
EOC: Epithelial ovarian cancer
FSS: Fertility-sparing surgery
OS: Overall survival
RS: Radical surgery
